# Transfer of Learning between Hemifields in Multiple Object Tracking: Memory Reduces Constraints of Attention

**DOI:** 10.1371/journal.pone.0083872

**Published:** 2013-12-11

**Authors:** Mark Lapierre, Piers D. L. Howe, Simon J. Cropper

**Affiliations:** Melbourne School of Psychological Sciences, The University of Melbourne, Parkville, Victoria, Australia; University of Akron, United States of America

## Abstract

Many tasks involve tracking multiple moving objects, or stimuli. Some require that individuals adapt to changing or unfamiliar conditions to be able to track well. This study explores processes involved in such adaptation through an investigation of the interaction of attention and memory during tracking. Previous research has shown that during tracking, attention operates independently to some degree in the left and right visual hemifields, due to putative anatomical constraints. It has been suggested that the degree of independence is related to the relative dominance of processes of attention versus processes of memory. Here we show that when individuals are trained to track a unique pattern of movement in one hemifield, that learning can be transferred to the opposite hemifield, without any evidence of hemifield independence. However, learning is not influenced by an explicit strategy of memorisation of brief periods of recognisable movement. The findings lend support to a role for implicit memory in overcoming putative anatomical constraints on the dynamic, distributed spatial allocation of attention involved in tracking multiple objects.

## Introduction

Many tasks involve visually keeping track of more than one moving object in the scene. These include driving, watching a film, or simply walking through a crowd. Furthermore, some activities, such as playing sports or video games, require that individuals adapt to changing or unfamiliar conditions to be able to perform well. Such adaptation has been demonstrated indirectly in the superior tracking performance of Officer Training Corps members [1] and radar operators [2] compared to untrained participants. Experienced action video game players show similarly superior performance in general, and the act of playing action video games has been shown to improve tracking performance in participants who had little or no experience playing those games [3]. 

**Figure 1:**
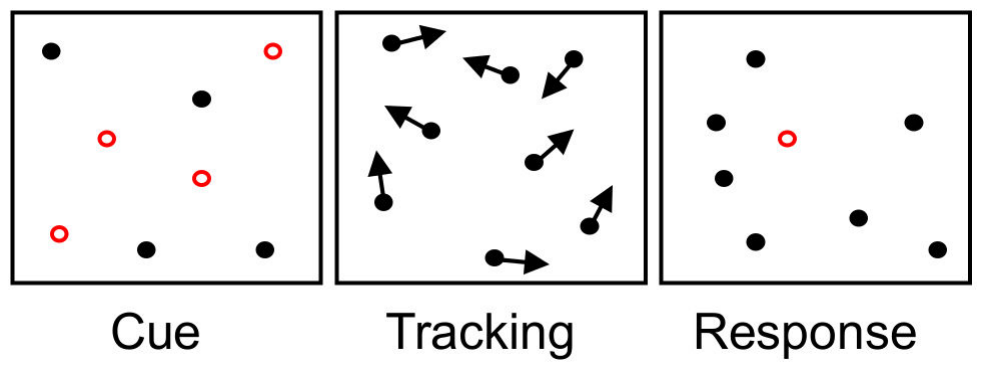
An example of a typical MOT task. The targets to be tracked are indicated during the cue phase, e.g., by flashing red for 1.5 seconds. The cues disappear and all objects begin moving independently during the tracking phase for a limited time, e.g., 6 seconds. Finally, during the response phase, the observer is prompted to indicate the location of the targets; in this example one object is probed and a ‘yes’ or ‘no’ response would be made. The square boundaries are shown only for illustrative purposes; the stimuli are usually bounded by the screen dimensions.

Typically, tracking performance is evaluated using a specifically developed Multiple Object Tracking (MOT) task; a computer-based task requiring sustained, distributed attention directed towards simple, dynamic, highly controlled, visually identical stimuli [4]. In this task, participants are required to keep track of a subset of moving objects, for example four of eight identical discs (Figure 1). At the start of each trial the four target discs are briefly cued, in this case by being coloured red. The cues disappear as the discs begin to move along random trajectories within an imaginary square for several seconds. Once the discs stop moving tracking accuracy is measured; for example, one object might be probed (e.g., coloured red) and the participant would indicate if it was a target or not. MOT tasks have predominantly been used to study attention [5]; however, they have also provided evidence of a role for visual short-term memory in tracking, showing that tracking improves when objects are visually unique [6-8], and degrades when performed concurrently with a task that requires visual short-term memory [6,9,10]. One model of tracking argues that long-term memory also contributes to tracking by facilitating the process of binding each target to its location [11]. While tracking clearly involves aspects of both attention and memory, it is unclear how those processes change or interact when tracking performance improves either through specific or non-specific training.

**Figure 2:**
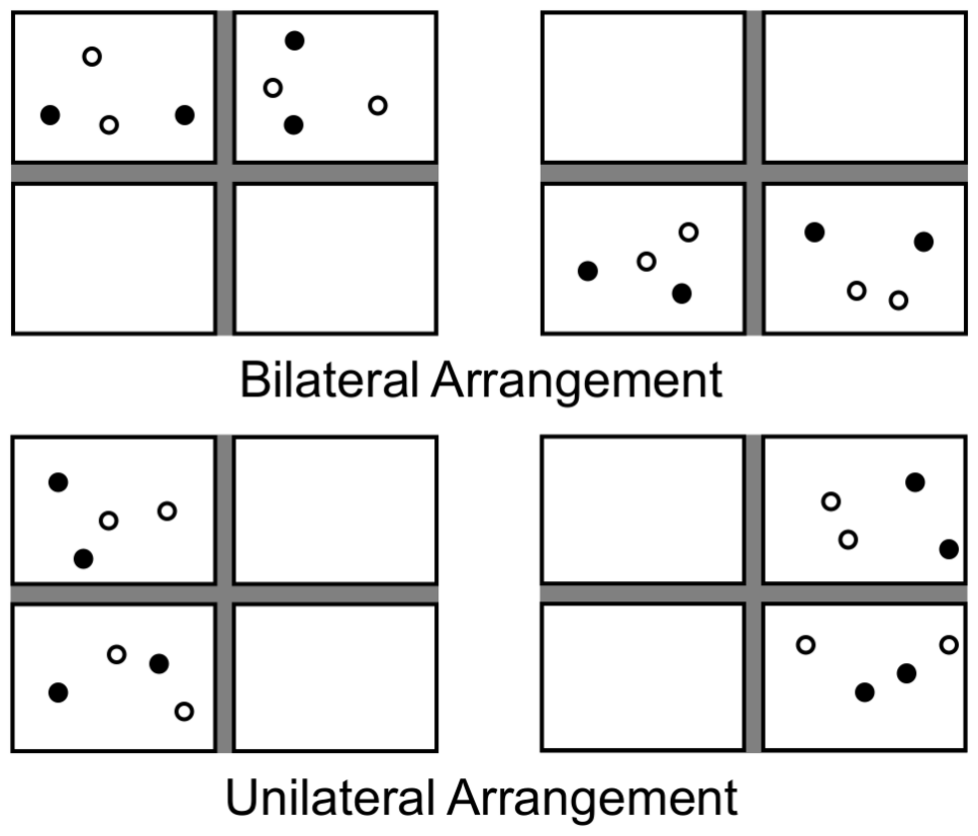
A diagram of the arrangement of stimuli in the initial hemifield independence *experiment.* The initial hemifield independence experiment [12] found poorer performance when tracking targets constrained to the left or right visual hemifield (unilateral arrangement) compared to targets constrained to the top or bottom visual hemifield (bilateral arrangement).

It has been suggested that tracking resources are distributed independently across left and right visual hemifields [12], although the effect of such independence may not be observed if the task is dominated by memory processes [13,14], thus indicating an interaction between attention and memory, and one that might be present in MOT. To elaborate, the initial demonstration of hemifield independence involved an adaptation of a typical MOT task that divided the display into quadrants, with an equal number of objects constrained to each quadrant. Two quadrants were displayed simultaneously such that objects were distributed either bilaterally or unilaterally (see Figure 2). When observers were required to track four targets distributed bilaterally, across the left and right visual hemifields, performance was better than when required to track four targets within a single hemifield. That is, if four objects were tracked, two in the left hemifield and two in the right, performance was better than if all four targets were in the same hemifield, either the left or the right, two above the fixation point and two below. The authors suggest that during tracking attentional resources are allocated separately to the left and right visual hemifields. This is similar to the proposal that distribution of resources of attention is anatomically constrained to quadrants of the visual field [15]. It was argued that anatomically separate regions of the visual system are involved in tracking objects in each quadrant of the visual field, and that this anatomical separation results in relatively less interference between attentional foci in separate quadrants, compared to foci within a quadrant. The neural basis for the hemifield independence effect has been investigated in a study involving transcranial magnetic stimulation [16]. When an observer’s left or right intraparietal sulcus (IPS) was temporarily inactivated using transcranial magnetic stimulation, tracking in the contralateral visual hemifield was significantly disrupted, but not completely eliminated as the hypothesis of full independence would predict. Under normal circumstances the right IPS is responsible for tracking objects in the left visual field and the left IPS tracks objects in the right visual field. The IPS in each hemisphere inhibits ipsilateral tracking by the IPS in the contralateral hemisphere. The researchers argued that it was the disruption to this contralateral inhibition that caused tracking not to be completely eliminated in the opposite hemifield. The disruption of contralateral inhibition allowed, for example, the right IPS to track targets in the left hemifield as normal, but to also track targets in the right hemifield, albeit at reduced capacity. Partial hemifield independence was also demonstrated in a replication [17] of the initial hemifield independence experiment [12], and also, in the same study, when tracking objects with visually unique identities. Finally, other researchers failed to find an effect of hemifield independence when there was only one target per hemifield, suggesting the effect may only occur at higher tracking loads [18]. 

A separate study also investigated hemifield independence, using change detection tasks rather than an MOT task [13]. It is pertinent because it examined hemifield independence of memory, as well as hemifield independence of attention. In one experiment observers were required to detect a change, if present, in the positions of a random array of squares. Performance was better when the squares were distributed between the left and right visual hemifields, compared to when they were presented within either the left or right hemifield. On the other hand, when observers were asked to detect a change in the colour of squares, rather than in their position, performance was similar whether the squares were all presented within either the left or right hemifield, or were distributed between both hemifields. These findings lend support to the speculation that hemifield independence during tracking may be the result of independence of attentional resources at an early selection stage, whereas later processing stages, such as identification and memory storage, are not hemifield independent [12,13]. 

The notion of differing patterns of hemifield independence of attention versus memory has been incorporated into a model of MOT [17]. The model was developed to account for tracking objects with unique identities as well as those that are visually identical. It proposes a two-stage process in which the first stage is responsible for tracking, with each hemisphere contributing to tracking in both visual hemifields, but at reduced capacity for the ipsilateral hemifield. The second stage binds each object’s identity to its location as supplied by the first stage. Since the second stage involves identity processing, the hemifield independence effect is reduced as a result of the relatively less error-prone functioning of the second stage compared to the first stage. These findings suggest that attention and memory processes interact in response to task demands, altering the degree to which putatively attention-driven effects are apparent. If this is the case, it is possible that greater involvement of memory processes would reduce or eliminate the hemifield independence effect, even when tracking visually identical objects. 

While MOT tasks have typically been used to study attention [5], they have also been used in the context of learning, allowing evaluation of particular interactions between attention and memory. For example, tracking performance in an MOT task was found to improve in response to repeated presentations of unique object motion paths, i.e., unique target and distractor trajectories [19]. However, no learning was found if the trajectories were manipulated under two conditions: (1) when the objects that were initially learned as distractors were subsequently tested as targets (i.e., targets and distractors were switched) and (2), when half of the objects learned as targets were tested as distractors, while half of the objects learned as distractors were tested as targets (i.e., targets and distractors were mixed). Ogawa, Watanabe, and Yagi [20] found similar results; tracking performance improved as observers repeatedly saw the same trajectories over multiple blocks, however, less improvement was found if only target trajectories were repeated (i.e., distractor trajectories were randomised). Repetition of distractor trajectories alone did not result in performance improvement. Further, when previously learned distractors were tested as targets, performance was worse than for unlearned trajectories, suggesting learned inhibition of distractor trajectories. The authors suggested these findings demonstrate the implicit encoding of dynamic global spatiotemporal relationships. Finally, when a learned trajectory was shown in reverse, performance was equivalent to when the learned trajectory was played in the correct order, suggesting that this is a form of associative learning in which backward and forward predictions are comparable, and that temporal prediction is not integral to attentive tracking [19]. 

Some aspects of learning in MOT may be understood in the context of statistical learning. Statistical learning involves the automatic and unconscious encoding of spatiotemporal statistical regularities [21,22]. Since its initial demonstration in audition [21], statistical learning has subsequently been shown in vision [22,23]. Similarities have been noted between learning in MOT, and implicit, statistical learning in the form of contextual cueing [19,20]. Contextual cueing is a learning effect that occurs when regularities in the configuration of a visual display aid deployment of attention within that display. The initial contextual cueing experiments involved a search task using a static display [24], although a similar effect has been shown using a dynamic display [25]. Regarding the aforementioned similarities between learning in MOT and statistical learning, Ogawa and colleagues [20] demonstrated tracking improvement in response to repeated presentations of the same trials, allowing the researchers to conclude that implicitly learned dynamic configurations of targets and distractors facilitate tracking, similar to how implicitly learned configurations of targets and distractors facilitate search; i.e., learning in MOT is a contextual cueing effect. Importantly, statistical learning has been shown to occur in the absence of awareness, but only in the presence of attention [22,26]. Thus it involves an interaction between attention and implicit memory, i.e., attention-gated learning of which the individual is unaware.

Perceptual learning may also play a role in MOT. Perceptual learning is generally understood to involve changes to the perceptual system that are relatively long lasting [27], and usually specific to stimulus features [28]. Perceptual learning differs from statistical learning in that the latter involves learning probabilistic spatiotemporal relationships between stimuli, while the former involves learning specific details about particular stimuli under particular conditions. Statistical learning extracts and encodes statistical regularities of stimuli, e.g., learning particular sequences of letters in an apparently random stream. On the other hand, perceptual learning improves the extraction and processing of perceptual information that stimuli contain, e.g., learning to detect and identify particular faces obscured by noise. A detailed discussion of the diverse aspects of perceptual learning research can be found in numerous extensive reviews [29-38]. One of the recurring findings of perceptual learning research is that performance improvement is specific to the location in which stimuli are presented during training [28].

The aim of this study was to further investigate the interaction of attention and memory in MOT. Specifically, to determine if hemifield independence is related to the relative dominance of processes of attention versus memory, and if putative anatomical constraints on attention prevent between-hemifield transfer of learned representations of trajectories. If reduced hemifield independence is related to dominance by memory processes, then introducing such an overt element of memory processing to the task was expected to allow hemifield independence to be breached, at least to some extent. Further, if the learning involved is a form of statistical learning, not only should what is learned in one hemifield improve performance in the opposite hemifield, but performance may not be related to recognition of what is learned. However, if the learning is a form of perceptual learning, performance should improve only in the hemifield in which stimuli appeared during training, but again may not be related to recognition of what is learned.

## General Methods

In each experiment observers were trained to track targets following sets of unique trajectories. Each trajectory was confined to one of four quadrants, with two quadrants displayed simultaneously in either the left or the right visual hemifield. Following training the observers were then tested to determine if tracking performance had improved. Testing also evaluated if any improvement was still evident when the trained trajectories were displayed in the hemifield opposite to that in which they appeared during training. For training, a forward-chaining technique was used. Forward-chaining is a part-task progressive learning technique in which the task to be learned is segmented, and the segments are learned individually, in order [39], as in to learning to play the first few bars of a piece of music before attempting subsequent bars. Such a technique can result in more efficient learning and has been used in a variety of tasks including mock submarine systems control and navigation [40] and piano playing [41].

### Participants

Participants of each experiment were colleagues of the authors, or volunteer undergraduate students who received a small monetary reimbursement ($15) for their participation. All participants provided informed written consent and the study was approved by the Department Human Ethics Advisory Group in the Melbourne School of Psychological Sciences at the University of Melbourne. All participants had normal or corrected to normal vision.

### Apparatus

The participants viewed the stimuli on a 21-inch CRT monitor at a resolution of 1600 by 1200 pixels with a frame rate of 100 Hz at a distance of 68 cm. Stimuli were presented in MATLAB [42] using the Psychophysics toolbox [43,44].

### Stimuli

**Figure 3:**
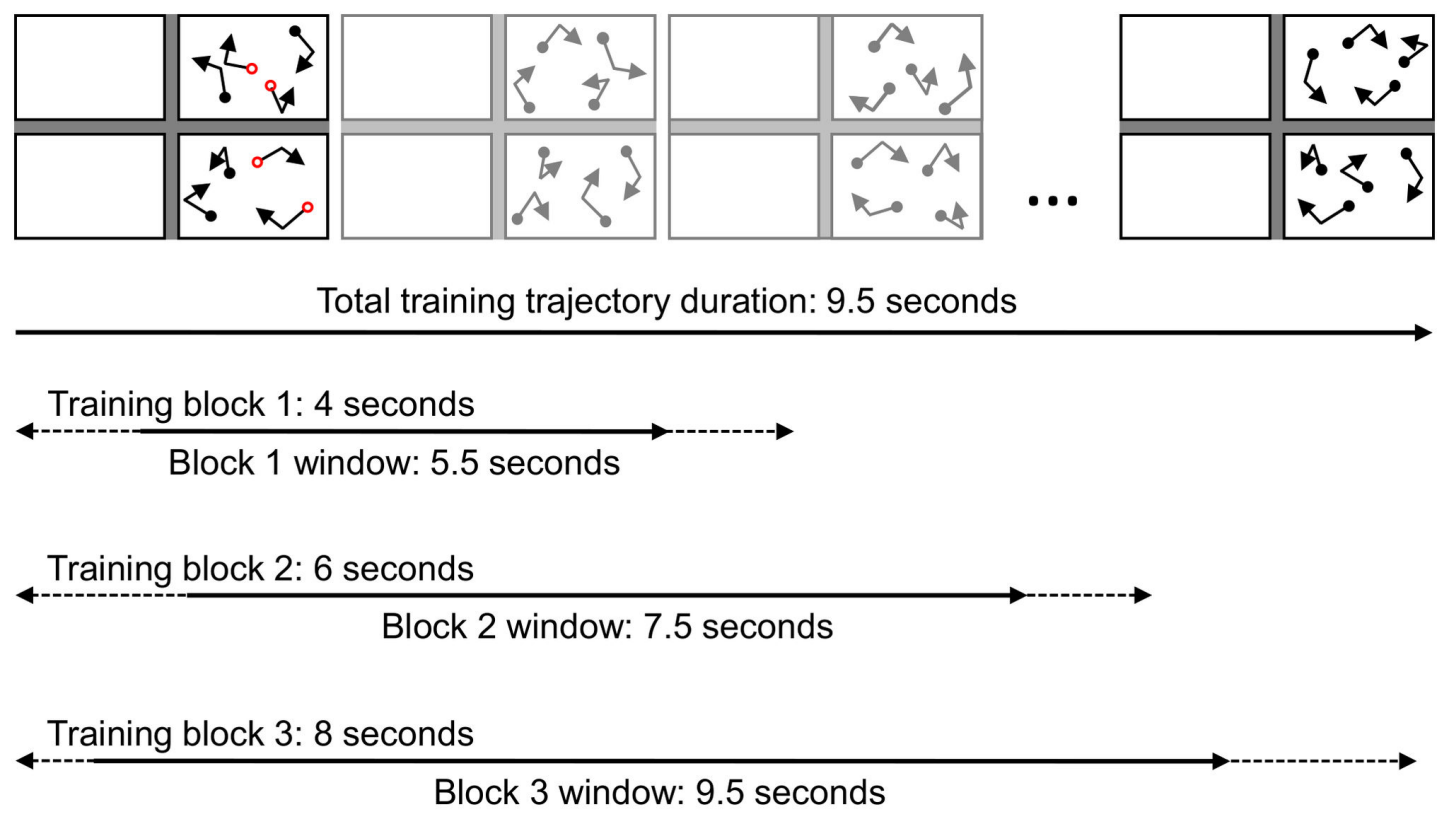
An example of the timing of training trials for each set of trajectories. Successive segments of the full trajectory set were chained to the end of previous segments; thus, this style of training is referred to as forward-chaining. Training proceeded from one segment to the next once an observer could track at least 80% of targets averaged over the previous 15 trials (or 5 trials during the first block), or once a segment was repeated 25 times. Dashed lines represent the block window; the portion of the original trajectory set from which trials in the training block were sampled. Solid lines represent examples of trials from the training blocks.

**Figure 4:**
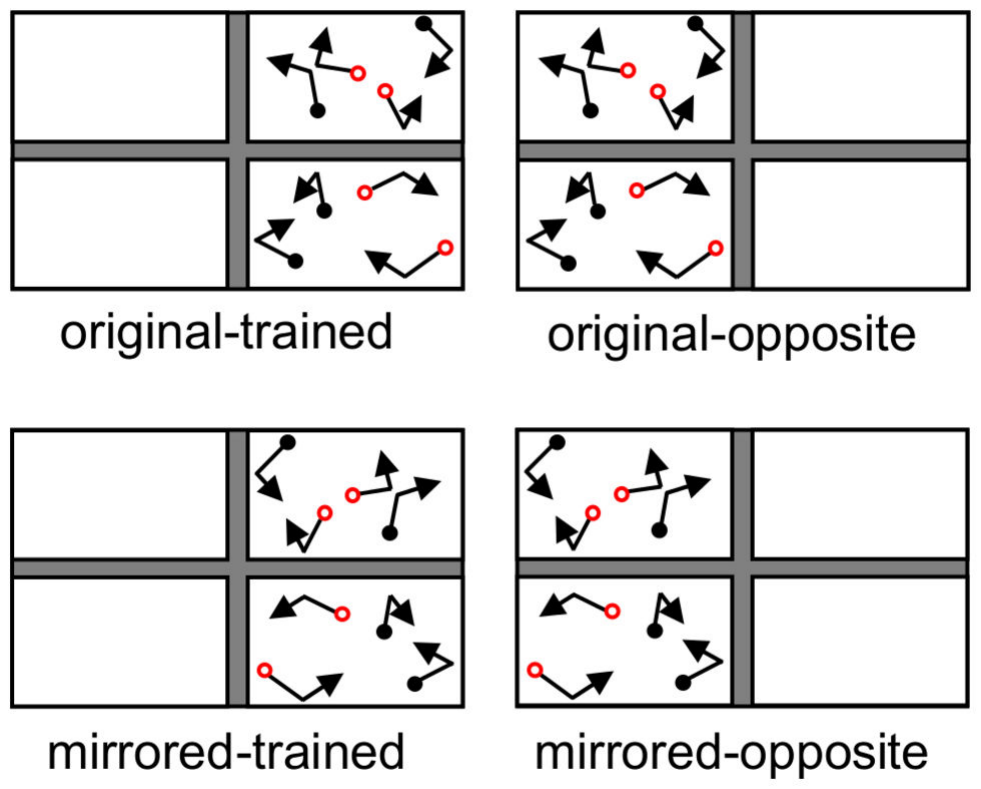
Test conditions comprised of spatial manipulations of the training trajectory sets. Trials in the original-trained and mirrored-trained conditions were 6 second portions of the 9.5 second trained trajectory sets and their mirrored versions, displayed in the hemifield in which they appeared during training. Trials in the original-opposite and mirrored-opposite conditions were portions of the same trained trajectory sets, but were displayed in the hemifield opposite to that in which they appeared during training. Targets are coloured red, distractors are coloured black.

The stimuli were comprised of 8 discs of 1 degree of visual angle (°) in diameter displayed on a white background. The CIE co-ordinates of the point of the background were x = 0.271, y = 0.284. The region in which the discs could be displayed subtended 29° by 21°. This region was divided into 4 quadrants subtending 14° by 10°. Each quadrant was separated from its neighbours by grey bar of 1° in width for the vertical separation, and 1° in height for the horizontal separation. A black fixation cross was displayed in the centre of the screen, subtending 0.5° by 0.5°. The luminance of the white background, grey inter-quadrant separators, and black fixation cross were, respectively, 33.3 cd/m^2^, 5.7 cd/m^2^, and 0.2 cd/m^2^. In any given trial the 8 discs would be constrained to move within 2 quadrants, 4 discs per quadrant, of which 2 were targets and 2 were distractors. Quadrants were arranged unilaterally, i.e., both were either left or right of fixation, one below the horizontal midline and one above (see Figure 3 and Figure 4 for examples). Discs were not permitted to collide; whenever a disc came within 2.5° of the centre of another disc, the direction of both discs was changed by adding the current motion vector to a vector directed away from the colliding disc. Discs also bounced off the walls of the quadrant in which they were constrained. Each trial sequence began with a cue phase during which the 4 target discs appeared red while the remaining distractor discs appeared black. Discs were stationary for 1 second after which the tracking phase began, with discs moving in random directions at a constant speed. After another 1.5 seconds of movement the red target discs became black, identical to the distractors. The discs continued to move for a few seconds before stopping. The exact duration differed between experiments and phases of each experiment as detailed below. The stimuli displayed during the subsequent response phase depended on the stage of the experiment and are also described below. 

### Procedure

Each experiment was conducted over multiple sessions for each observer, with each session lasting between 45 minutes and one hour. During each session observers were trained to track discs following four unique patterns of movement, i.e., four unique sets of trajectories, and were tested on those same unique sets of trajectories. Each set contained eight trajectories, the trajectories of four target discs and four distractor discs; two targets and two distractors per quadrant (Figure 3). Unique sets of trajectories were pre-generated prior to each session. Pilot testing revealed inconsistent learning across sets of trajectories; some unique sets were readily learned while others resulted in no learning whatsoever. The cause was identified as variability in the difficulty of tracking due to idiosyncrasies in the movement of discs following particular trajectories. Specifically, as the eccentricity of discs increased, i.e., as they travelled further from fixation and into peripheral vision, losing a target during target-distractor collisions became more and more likely, thereby increasing tracking difficulty (for a demonstration of a similar effect, see Bettencourt and Somers [45]). Sets of trajectories with greater target eccentricity were harder to track, and made learning more difficult. To overcome this issue observers were trained to track a set of trajectories as well as a mirrored version of that set. Mirroring flipped the set about a vertical meridian through the hemifield, as if viewed in a mirror. Thus, a set of trajectories with a large average eccentricity would tend to have its average eccentricity reduced. Performance for original and mirrored versions of a set of trajectories was averaged to produce an estimate of accuracy less biased by effects of eccentricity. Further pilot testing revealed more consistent learning following this modification to the design. Pilot testing also suggested that the forward-chaining technique was at least as effective at improving performance as the unstructured, random-order presentation employed by previous researchers and also reinforced the importance of learning for the observers. 

Prior to training, each observer undertook a calibration phase to determine the speed at which discs should move for the observer to accurately track targets over approximately 75% of trials. Accuracy was measured via a probe response; during the response phase, while all discs were stationary, one disc appeared red and all others appeared black. The observer was asked to press the ‘y’ key if the probed disc was a target, or the ‘n’ key if it was a distractor. Calibration was a 2-stage process in which performance data was collected using the method of constant stimuli, and the QUEST algorithm [46] was used to estimate psychometric function slope and threshold. During the first stage trials were presented to the observer with discs moving at 5 speeds randomly and uniformly distributed over 50 trials, with speeds chosen to cover a wide range from chance to perfect performance, based upon the observers’ previous experience with MOT-like tasks. This ranged from a minimum of 5°/s to a maximum of 20°/s. The data from this stage were input into the QUEST algorithm to estimate the psychometric function slope, and to roughly estimate the 75% performance threshold. The second stage then presented the observer with another 50 trials at 5 speeds of a narrower range, centred on the roughly estimated 75% threshold. Finally, all 100 trials from both stages were plotted, outliers removed, and the remaining data submitted to QUEST to estimate a 70% threshold (lower than 75% to account for further improvement expected during the experiment). If the output from QUEST indicated excessive variance in the threshold estimate (>2 *SD*) stage 2 was repeated. 

During training each unique set of trajectories was learned separately and individually; one set of trajectories was learned before proceeding to the next. The implementation of forward-chaining involved segmenting each unique set of trajectories into 2 second portions (Figure 3). Performing an MOT task for 2 seconds is a relatively trivial task, so the first 2 segments were presented during the first block of training, with subsequent segments added during successive blocks. Thus, during the first training block 4 seconds of a trajectory were displayed, followed by 6 seconds in block 2, and 8 seconds in block 3. Each block was repeated until tracking performance reached a learning criterion of 80% correct averaged over the preceding 15 trials (or 5 trials during the first block), or until the block had been repeated 25 times. It was necessary to prevent observers from simply recognising the start or end positions of previous presentations of the same set of trajectories and thereby forgoing tracking. Therefore, the first frame of each trial was randomly chosen from among the frames of the first 1.5 seconds of the original set of trajectories. In combination with a consistent trial length, this was intended to ensure the start and end positions were unpredictable. The duration of each unique set of trajectories was 9.5 seconds in total, however during training a maximum of 8 seconds was displayed during any trial. The start and end frames were similarly staggered during testing; the first frame of each trial was set to a random frame from within the first 3.5 seconds of the original set of trajectories, and the trial length was 6 seconds. This was intended to test learning of the majority of the full 9.5 seconds of the set, although the training design would reinforce learning of the earlier portion to a greater extent than later portions. Throughout the training and testing phases tracking performance was measured via full report; during the response phase of each trial all discs were displayed coloured black and observers were asked to click on each target. After 4 discs were selected any correctly selected targets became green, while any unselected targets became red. 

Learning was evaluated in a test phase comprised of 5 blocks. Test trials included 6-second portions of trained trajectory sets, and untrained, randomly generated trajectory sets that were also of 6 seconds in duration. Test trials that were based on trained trajectory set portions were created by setting the first frame of the test trial to be a randomly selected frame within the first 3.5 seconds of the trained trajectory set. These training-derived test trials were displayed in the hemifield in which they were displayed during training (the *original-trained* and *mirrored-trained* conditions), or in the opposite hemifield (the *original-opposite* and *mirrored-opposite conditions*). See Figure 4. Untrained, *new*, trials were counterbalanced across hemifields, and were matched to the number of trials in each original/mirrored pair of training-derived trials. Thus, in each test block there were 2 trials in each of the original-trained, original-opposite, mirrored-trained, and mirrored-opposite conditions, and 8 new trials (4 per hemifield). Trials were randomly intermixed within each block.

### Data analysis

In each experiment analyses were conducted in accordance with Fisher’s procedure as described by Levin, Serlin, and Seaman [47]. Specifically, a one-way ANOVA was conducted to test for an effect of training on condition (trained, opposite, or new). If the test was significant all pairwise comparisons (*t*-tests) were performed at the alpha level of the omnibus test (.05). If the ANOVA was not significant, it was assumed there was no difference between conditions and planned comparisons were not conducted. Levin and colleagues demonstrate that in the specific case of comparisons between three conditions, Fisher’s procedure ensures that the family-wise error cannot exceed the prescribed alpha level.

## Experiment 1

This experiment served as the initial test of the main hypothesis of this study. If hemifield independence effects are reduced by the relative dominance of memory processes over processes of attention, learned trajectories displayed in the opposite hemifield should be tracked better than new trajectories. 

### Participants

There were seven participants (5 male, 2 female), one of whom was the first author (M.L). Two participants were volunteer undergraduate students. The remaining participants were colleagues of the authors. Two participants (M.L. and R.L.) had prior experience with MOT tasks; the remainder were inexperienced observers. The data for one participant were excluded from analysis due to repeated failure to reach the prescribed learning criterion. 

### Results

**Figure 5:**
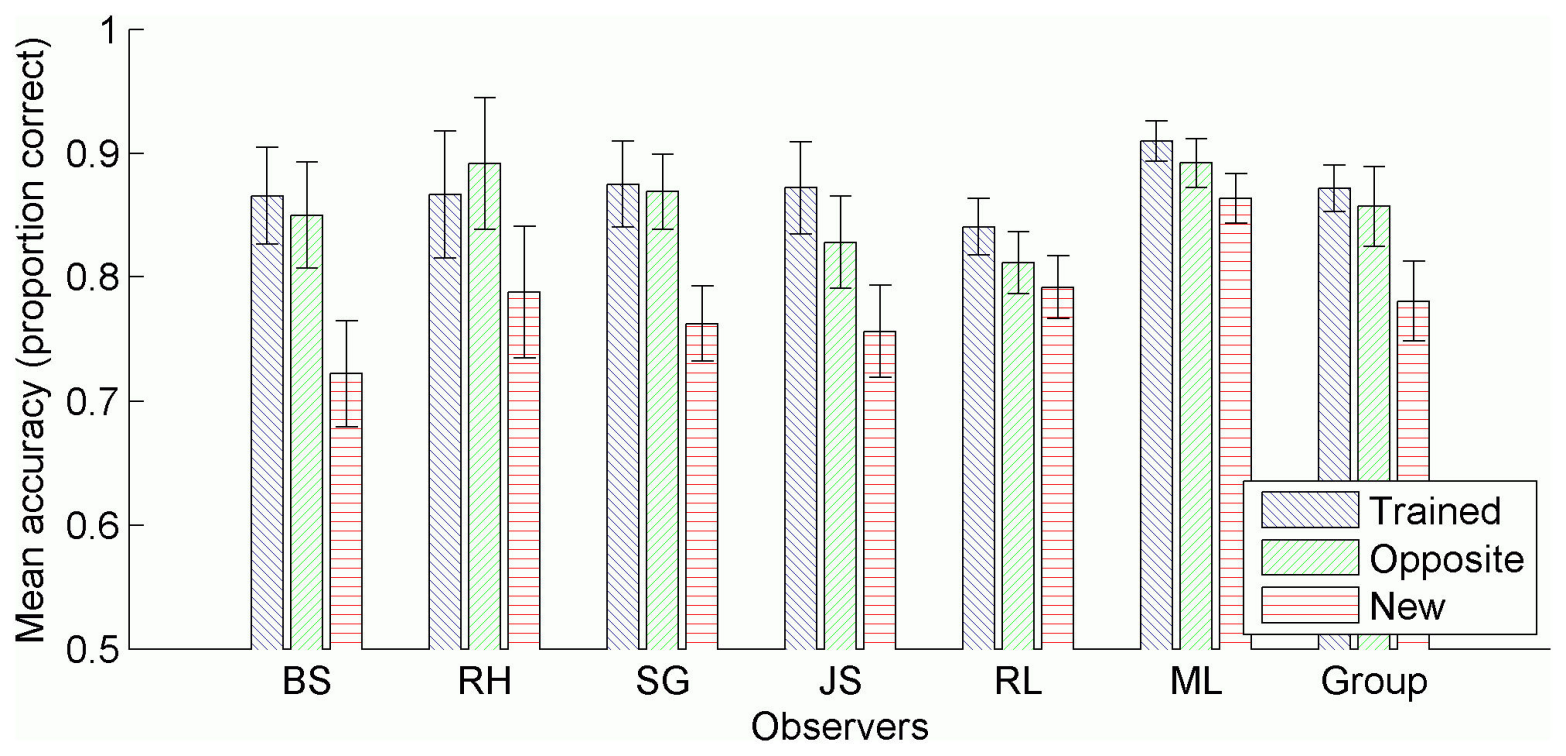
Mean tracking accuracy in each condition. Accuracy measured as the proportion of targets tracked, averaged across trials in each condition, separately for each observer. Group means also included. All error bars represent 95% confidence intervals, and were calculated as per Morey [64] for the group data.

Data analysed here as the *trained* condition is comprised of responses to the original-trained and mirrored-trained conditions of the test phase. Similarly, data analysed here as the *opposite* condition is comprised of responses to the original-opposite and mirrored-opposite conditions. Responses were also collected for trials in the untrained, *new* condition. Figure 5 shows the tracking accuracy in each condition for each of the 6 observers, as well as combined data for the whole group. A one-way ANOVA was conducted separately for each observer to determine the effect of condition on tracking accuracy (Table 1). Observers B.S., R.H., S.G., and J.S. all showed similar performance across trained and opposite conditions (all *p*’s >.05), and greater performance in those conditions compared to the new condition. The group analysis reflects the same pattern of results. Observers R.L. and M.L. each also showed statistically similar performance in the trained and opposite conditions, however, compared to the new condition, each achieved greater performance only in the trained condition but not in the opposite condition. Note that performance in the new condition for observer M.L. was considerably higher than the 70% untrained threshold specified during the calibration phase. M.L. performed many sessions at the commencement of the experiment for which the data were discarded due to problems with the data collection software. This resulted in greater performance improvement beyond initially calibrated levels than expected. Thus it is possible that the data for the trained and opposite conditions are limited by a ceiling effect, underestimating the true size of the learning effect for observer M.L. 

**Table 1:** Results of ANOVA and Planned Comparisons for each Observer for the Effect of Hemifield Display Relative to Training.

Observer	ANOVA Results			Trained vs. Opposite			Trained vs. New			Opposite vs. New		
	*F *	*df *	*p *	*t *	*df *	*p *	*t *	*df *	*p *	*t *	*df *	*p *
B.S.	15.33	2,237	<.001	.57	158	.56	4.88	158	<.001	4.47	158	<.001
R.H.	9.08	2,237	<.001	.33	158	.74	3.64	158	<.001	3.49	158	<.001
S.G.	13.27	2,237	<.001	.24	158	.81	4.79	158	<.001	4.37	158	<.001
J.S.	8.68	2,237	<.001	1.53	158	.13	4.34	158	<.001	2.51	158	.013
R.L.	3.15	2,717	.043	1.42	478	.16	2.57	478	.01	1.07	478	.27
M.L.	5.42	2,717	.005	1.46	478	.14	3.29	478	.001	1.8	478	.07
Group	21.20	2,10	<.001	1.53	5	.19	5.66	5	.002	4.22	5	.008

### Discussion

The data suggest all observers learned to track their trajectory sets, however, four observers (B.S., R.H., S.G., and J.S.) demonstrated full transfer of learning between hemifields, while the remaining two observers (R.L. and M.L.) demonstrated only partial transfer. It is worth noting that the latter two observers had extensive prior experience with MOT tasks, while the former four did not. It is possible that the inexperienced observers appeared to show learning and transfer because they were required to track discs moving at a slower speed than more experienced observers, and that this slower speed produced final positions of the targets that did not vary substantially, even though the start (and thus end) point of each test trial was varied from trial to trial within a 1.5 second window. This effect of experience may also be the result of the effect of task difficulty on the specificity of perceptual learning; learning effects become more likely to generalise beyond a trained location as task difficulty decreases [48]. Assuming the learning effects are at least partly representative of perceptual learning, the relatively faster moving discs tracked by experienced observers may have resulted in a harder learning task due to the increased complexity of patterns of movement, and thus reduced transfer compared to that shown by the inexperienced observers. The following experiment aimed to confirm if learning and transfer between hemifields still occurred after ensuring that evidence of learning and transfer could not be a result of predictable final target positions, and that the speed of object movement was similar for all observers. 

## Experiment 2

This experiment was designed to test the hypothesis that the results of the previous experiment were due to objects moving faster for the experienced observers. Because each trial ended at a random time, the faster a disc moves, the harder it is to estimate its final location. To avoid this potential confound, in this experiment the tracking task was calibrated for each observer individually, by adjusting set size, disc size, and the minimum distance between the discs so that speeds of the disc movement were equivalent between observers, similar to the speed at which inexperienced observers tracked the discs in the first experiment. 

### Participants

There were six participants (all male), one of whom was the first author (M.L.). Four participants were volunteer undergraduate students (B.S., Y.N., J.S., and D.B.). The remaining participant was a colleague of the authors (R.L.). Four observers (B.S., M.L., J.S., and R.L.) participated in the previous experiment, while the rest were inexperienced observers. 

### Stimuli

Experienced observers (M.L. and R.L.) tracked 4 targets among 8 distractors; 2 more distractors per quadrant than in the previous experiment (i.e., in each quadrant there were 2 targets and 4 distractors). This change was made to allow the speed of movement for experienced observers to be reduced to a level similar to that of inexperienced observers, but without making the task too easy. Stimuli parameters are shown in Table 2.

**Table 2:** Stimuli Parameters for each Observer.

Observer	Speed (°/s)	Disc diameter (°)	Minimum separation (°)	Distractors per quadrant
M.L.	9	0.7	1.8	4
R.L.	9	0.7	1.4	4
D.B.	7	1.0	2.5	2
B.S.	9	0.6	1.4	2
Y.N.	8	1.0	2.0	2
J.S.	7	1.0	2.0	2

### Procedure

The initial speed was set at a level midway between the average of experienced and inexperienced observers who participated in the previous experiment (9°/s). If the method of constant stimuli and the initial QUEST procedure indicated that the speed at which an observer would track successfully 70% of the time differed from the initial speed by 2°/s or more, another parameter was altered and the process was repeated. If the observer’s predicted threshold speed was at least 5°/s greater than the initial speed, the number of distractors was increased by one. If the observer’s predicted threshold speed was between 2°/s and 5°/s greater than the initial speed, the disc size and the minimum distance between discs were alternately decreased by 0.1° until the predicted threshold speed reached the initial speed. The minimum distance between discs was never permitted to be less than double the disc diameter. No observer’s predicted threshold speed was lower than the initial speed by more than 2°/s. The duration of each full training trajectory was increased to 10 seconds, while the duration of each test trial was reduced to 5 seconds. 

### Results

**Figure 6:**
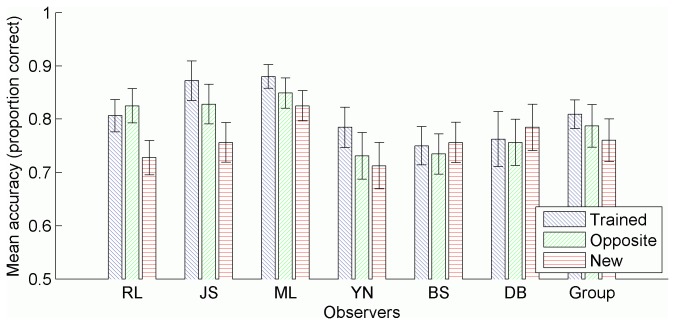
Mean tracking accuracy in each condition. Accuracy measured as the proportion of targets tracked, averaged across trials in each condition, separately for each observer. Group means also included. All error bars represent 95% confidence intervals, and were calculated as per Morey [64] for the group data.

Figure 6 shows the tracking accuracy of each observer in each of the trained, opposite, and new conditions. A one-way ANOVA was conducted separately for each observer to determine the effect of condition on tracking accuracy (Table 3). Observers R.L. and J.S. showed similar performance across trained and opposite conditions, and greater performance in those conditions compared to the new condition. Observers M.L. and Y.N. showed statistically similar performance in trained and opposite conditions (although the difference approached significance), however, compared to the new condition, they achieved greater performance only in the trained condition but not in the opposite condition. Observers B.S. and D.B. showed equivalent performance across all three conditions, a pattern of results similar to the group analysis, although the effect of condition approached significance in the group analysis.

**Table 3:** Results of ANOVA and Planned Comparisons for each Observer for the Effect of Hemifield Display Relative to Training.

Observer	ANOVA Results			Trained vs. Opposite			Trained vs. New			Opposite vs. New		
	*F *	*df *	*p *	*t *	*df *	*p *	*t *	*df *	*p *	*t *	*df *	*p *
R.L.	10.54	2,597	<.001	.84	398	.4	3.51	398	<.001	4.25	398	<.001
J.S.	8.68	2,237	<.001	1.53	158	.13	4.34	158	<.001	2.51	158	.01
M.L.	4.66	2,597	.009	1.87	398	.06	2.97	398	.003	1.26	398	.21
Y.N.	3.16	2,237	.04	1.85	158	.07	2.44	158	.02	.061	158	.54
B.S.	.4	2,237	.67									
D.B.	.35	2,237	.71									
Group	3.60	2,10	.07									

### Discussion

The results indicate that many, although not all, inexperienced observers failed to achieve significant learning, let alone transfer, while experienced observers continued to show learning and at least partial transfer. These results do lend support to the possibility that some observers relied on learning the final positions of discs, and were therefore unable to achieve adequate learning when the final positions varied to a greater extent. Specifically, observer B.S. showed no learning in this experiment, despite showing considerable tracking improvement in Experiment 1. As the only substantive difference in stimuli parameters between experiments for B.S. was a decrease in the length of test trials relative to training trajectory length, we conclude that in this experiment B.S. was unable to use the strategy of remembering the final positions of discs, a strategy which had served him well in the previous experiment. This may also apply to observer D.B., however we can only speculate since he did not perform Experiment 1.

These data also demonstrate that learning is possible without a strategy of remembering the final positions of objects. A likely alternative strategy could involve the learning of snapshots, i.e. brief periods of recognisable movement, but throughout a trajectory set rather than limited to the end. Author M.L. can report he did use such a strategy, and debriefing with R.L. revealed he did as well. It is possible that other observers could develop such a strategy spontaneously or through a directed attempt to learn more effectively. The varied patterns of learning and transfer may reflect individual differences in the ability to discern and use such strategies. For example, observer J.S. showed learning and full transfer in Experiments 1 and 2, despite not having experience with such an MOT task before. On the other hand, one observer was excluded from Experiment 1 since she was unable to reach the learning criterion during each session’s training phase. The following experiment was designed to investigate if a strategy of learning snapshots of movement can be learned, and if so, if it improves trajectory learning or transfer.

## Experiment 3

The aim of this experiment was to determine if learning was driven by recognition of snapshots. It has been suggested that representations of learned trajectories could not be moment-to-moment snapshots, as previous research had shown that four targets could not be learned in a static search task ([49], as cited in Makovski et al. [19]). However, the cited research did find that observers could learn four target locations, albeit after more training than required to learn a single target location [49]. Thus a snapshot-based account of representations involved in learning MOT trajectories should not be ruled out. 

If a strategy of remembering the final positions of a trajectory explains full transfer in the first experiment, but no learning in the second, then observers who show no or little learning are expected to show a greater ability to track and recognise portions taken from the end of a learned trajectory. In comparison, tracking and recognition of portions taken from the beginning or middle of a learned trajectory are expected to be worse. Conversely, observers who show learning and full or partial transfer are expected to accurately track and recognise snapshots distributed throughout a trajectory. Alternatively, if trajectory learning is not aided by a strategy of learning snapshots, there should be no relationship between evidence of learning (or transfer) and recognition of snapshots. 

### Participants

There were six participants (4 male, 2 female), one of whom was the first author (M.L). Two participants were volunteer students, however only one (Y.K.) completed the experiment; the other (S.T.) had to withdraw early for personal reasons. Two observers (M.L., and R.L.) participated in the previous experiment; one participated in the first experiment (S.G.). 

### Stimuli

All observers tracked 4 targets among 4 distractors, 2 targets and 2 distractors per quadrant. Remaining stimuli parameters were as shown in Table 4.

**Table 4:** Stimuli Parameters for each Observer.

Observer	Speed (°/s)	Disc diameter (°)	Minimum separation (°)
M.L.	15	0.5	1.3
R.L.	12	0.5	1.1
G.J.	8	1.0	2.7
S.G.	10	0.8	1.7
Y.K.	12	0.8	1.7

### Procedure

Observers performed the calibration and the training phase as per previous experiments, however, during training a snapshot capture block was performed after the training of each trajectory set. Observers were required to depress and hold down the space bar whenever they saw a brief pattern of movement (i.e., a snapshot) that they recognised. There were 5 identical snapshot capture trials per training trajectory set. A snapshot was measured as the duration the spacebar was depressed. A snapshot was considered reliable, and thus ‘valid’, if the snapshot durations overlapped for at least 3 of 5 trials. It was possible for no valid snapshots to be captured. 

During the test phase observers saw portions of the trained trajectories intermixed with random trajectories. Portions were 5s in length, i.e. half the length of a full trained trajectory. Targets were cued at the start of each portion. During the response phase of each trial observers made two responses: 1) a full report of targets as per previous experiments, and 2) a yes or no response to the question, “Did you recognise a snapshot?” Observers responded by pressing the ‘y’ or ‘n’. There were 5 test conditions. Trials from three were derived from trained trajectory sets. These were a *snapshot* condition in which portions included the snapshots the observer indicated during the snapshot capture phase, a *trajectory-end* condition in which portions ended within 0.5s of the end of the trained trajectory, and a *non-snapshot* condition in which portions began at least 1s after the start of a trajectory, or ended at least 1s before the end of a trajectory, and began and ended at least 0.5s before or after any snapshots, but could still contain snapshots at some point during the test phase, if an observer specified long or overlapping snapshots. Trials in each of the snapshot, trajectory-end, and non-snapshot conditions were displayed in the hemifield in which they appeared during training, as well as in the opposite hemifield. If, during the training phase, an observer had failed to identify a valid snapshot for a particular trajectory set, *snapshot* and *non-snapshot* conditions were not included, replaced by a *random-snapshot* condition in which the portion began at least 1s after the start of a trajectory, or ended at least 1s before the end of a trajectory. Trials in the *random-snapshot* condition were included to ensure a consistent number of trials per session if an observer failed to indicate a valid snapshot for one or more trained trajectories. Performance for the *random-snapshot* condition was not included in the analyses. The final condition was an untrained condition, referred to as the *new* condition, similar to the previous experiment, in which trajectories were randomly generated. 

### Results

**Figure 7:**
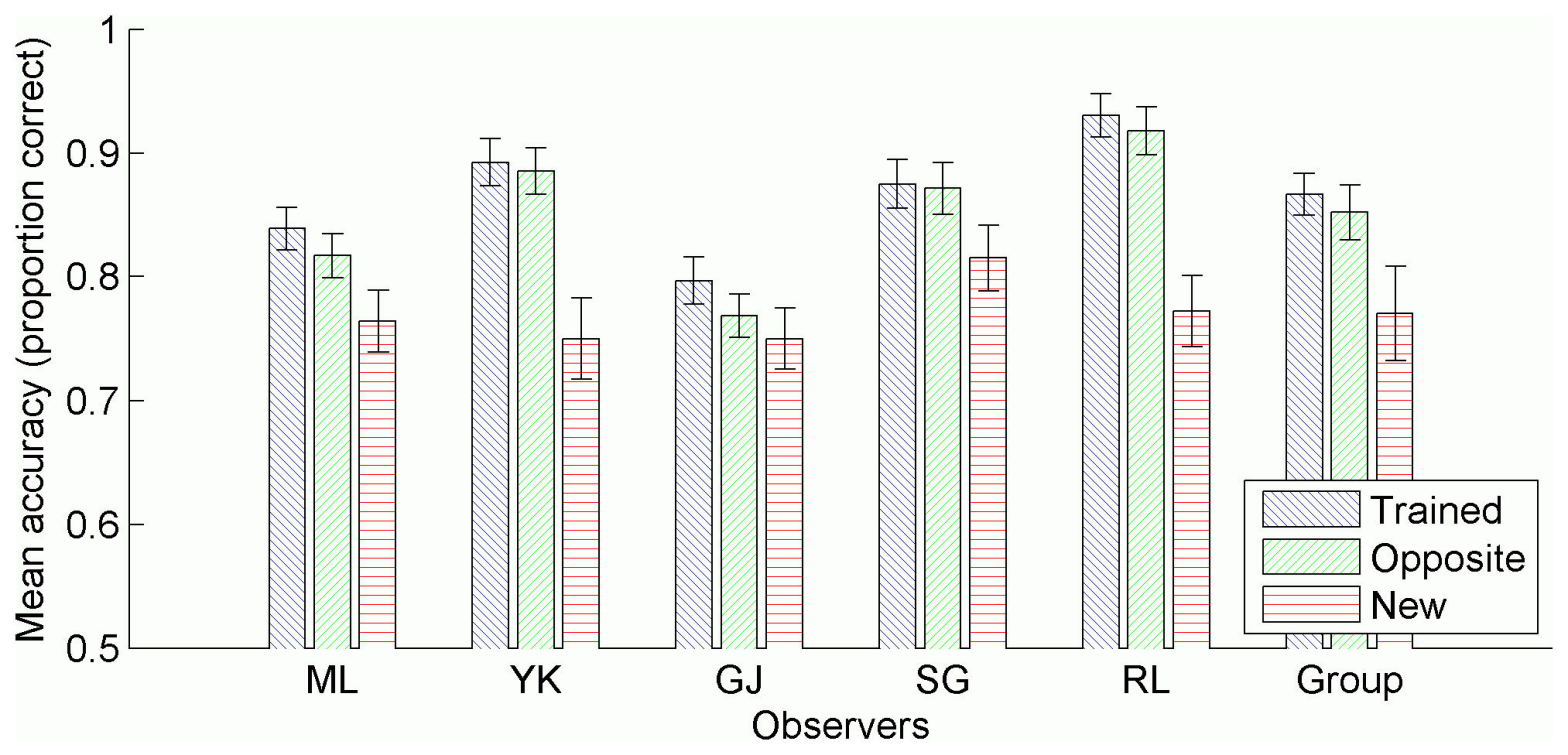
Mean tracking accuracy in each condition. Accuracy measured as the proportion of targets tracked, averaged across trials in each condition, separately for each observer. Group means also included. All error bars represent 95% confidence intervals, and were calculated as per Morey [64] for the group data.

A one-way ANOVA was conducted separately for each observer to determine the effect of training on tracking accuracy during testing, as per the previous experiments (Table 5). G.J. showed better performance in the trained condition compared to the opposite condition, and statistically equivalent performance in the opposite and new conditions. All other observers showed better performance for trained trajectory sets compared to those that were new, and equivalent performance whether the trained trajectory sets were displayed in the trained hemifield, or in the opposite hemifield. The group analysis reveals better performance in the trained condition compared to the opposite condition, and although significant, the difference is very small (Figure 7). 

**Table 5:** Results of ANOVA and Planned Comparisons for each Observer for the Effect of Hemifield Display Relative to Training.

Observer	ANOVA Results			Trained vs. Opposite			Trained vs. New			Opposite vs. New		
	*F*	*df*	*p*	*t*	*df*	*p*	*t*	*df*	*p*	*t*	*df*	*p*
M.L.	13.13	2,1053	<.001	1.75	790	.08	5	658	<.001	3.47	658	<.001
Y.K.	41.76	2,765	<.001	.51	574	.61	7.86	478	<.001	7.55	478	<.001
G.J.	5.1	2,735	.006	2.14	544	.03	2.99	463	.003	1.22	463	.22
S.G.	7.84	2,741	<.001	.25	550	.8	3.65	466	<.001	3.3	466	.001
R.L.	58.93	2,669	<.001	.94	478	.35	9.63	430	<.001	8.48	430	<.001
Group	14.01	2,8	.002	3.17	4	.03	4.27	4	.01	3.27	4	.03

**Figure 8:**
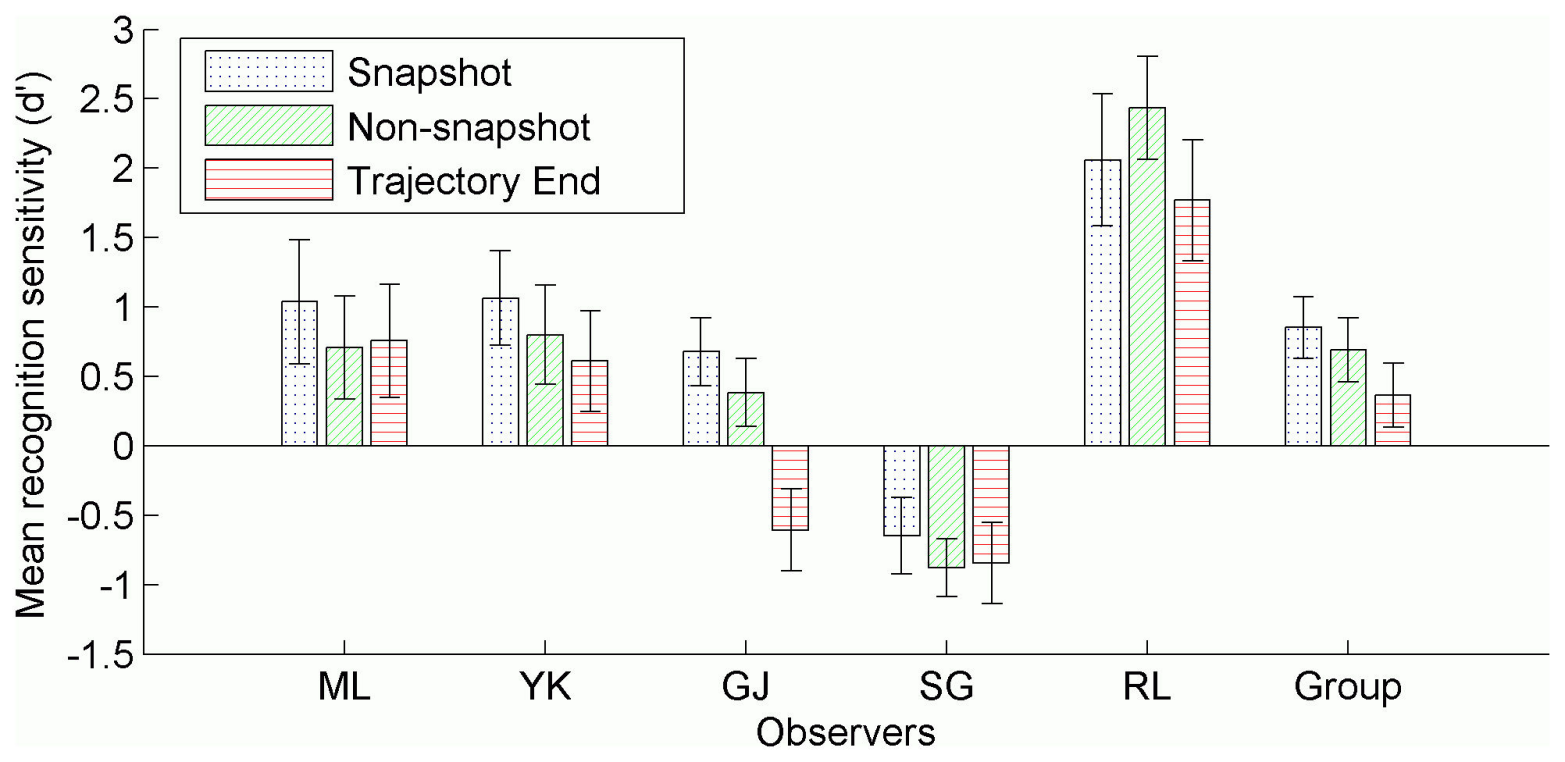
Mean recognition sensitivity in each condition. Recognition sensitivity (d’) by snapshot condition for each observer. Group means also included. All error bars represent 95% confidence intervals, and were calculated as per Morey [64] for the group data.

A one-way ANOVA was conducted separately for each observer to determine the effect of snapshot condition on tracking accuracy during testing, but no effect was found (all *p*’*s* > .05). This suggests that observers were not using snapshots to learn the trajectory sets; performance in the snapshot condition was not superior to performance for other portions of a trained trajectory set. This result was not because observers were unable to recognise the snapshots. An analysis of response bias and sensitivity separately revealed that this was not the case. These measures are derived from signal detection theory [50]. Sensitivity refers to the ability to distinguish between when a signal or target (in this case, a target disc) is present and when it is not. Response bias refers to a general tendency to respond either ‘yes’ or ‘no’. The sensitivity of snapshot recognition was measured using *d*’, calculated from observers’ responses as per Stanislaw and Todorov [51]. *d’ is* typically 0 or above, with 0 indicating chance performance, and positive numbers indicating increasingly accurate performance. Negative scores can result from measurement error or, for example, an observer accidentally responding ‘no’ when he or she intended to respond ‘yes’. Response bias was measured using *c* [52-54]. It was chosen partly because it is reasonably independent of sensitivity [53,55], but also because its interpretation is more straightforward than the more common β*. c* reflects the discrepancy, measured in standard deviation units, between an observer’s tendency to respond either ‘yes’ or ‘no’, and the neutral point at which there is no bias. A value of zero would indicate no bias, positive values indicated a bias towards responding ‘no’, and negative values indicate a bias towards ‘yes’. Analysis of *c* revealed a moderate (0.5 standard deviation units or greater) general tendency to respond ‘no’ that biased the raw recognition scores. The group data in Figure 8 suggest that sensitivity significantly differs between conditions, *F*(2,10) = 5.92, *p* = .02, being greater in the snapshot condition than in the trajectory-end condition. However, individual observer analyses show that the group performance is an artifact of poor recognition of portions of the end of a trajectory for observer G.J., and poor recognition overall for observer S.G. All other observers showed above chance recognition in each condition as measured by *d*’, with no difference between conditions (all *p*’*s* >.05). 

**Figure 9:**
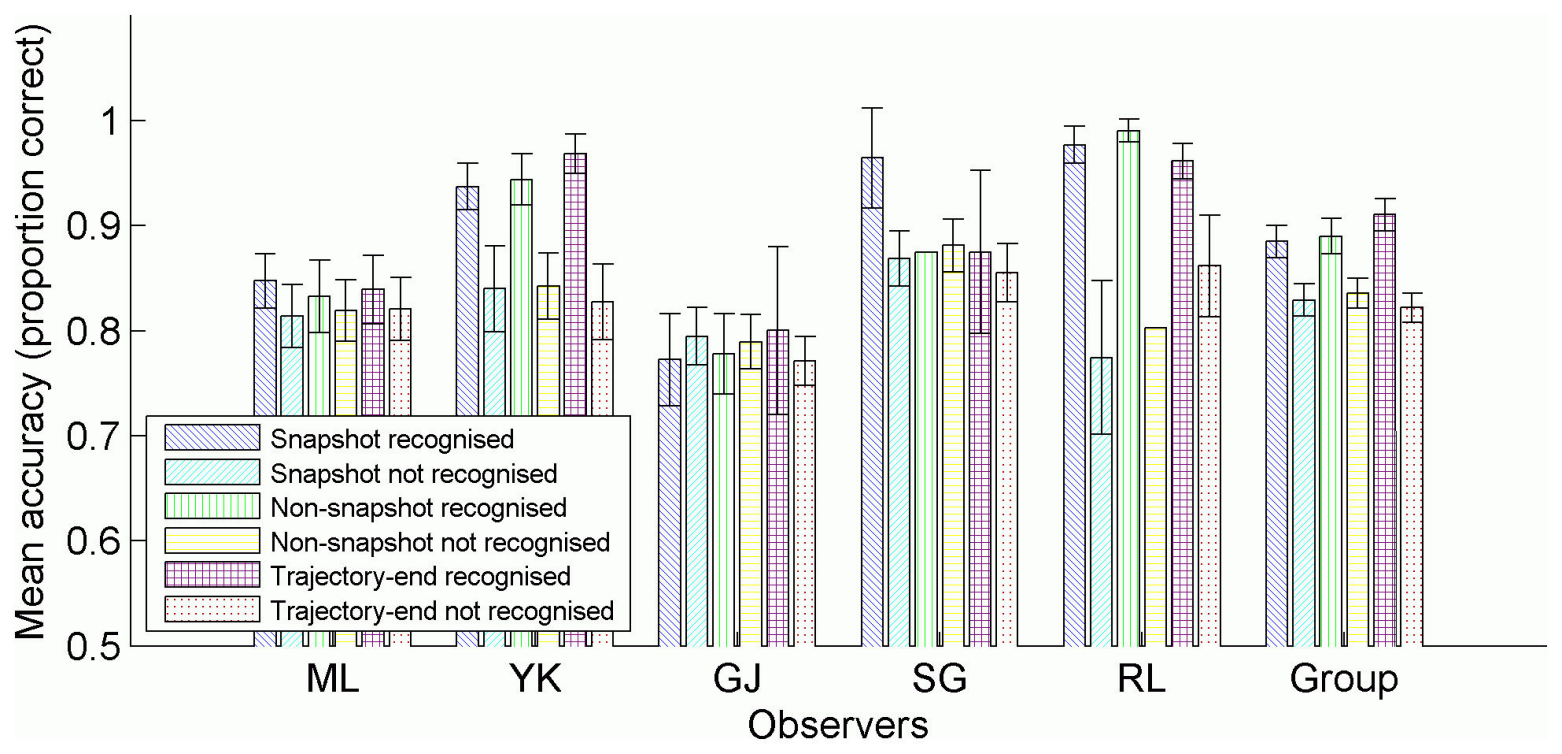
Mean tracking accuracy by snapshot condition and recognition. Accuracy measured as the proportion of targets tracked, averaged across trials in each condition, separately for each observer. Group means also included. All error bars represent 95% confidence intervals, and were calculated as per Morey [64] for the group data. Missing confidence intervals indicate conditions in which there were very few samples (<20).

Pearsons’ correlations between block means of tracking accuracy and recognition for all test phase conditions were between 0.3 and 0.75 (all *p*’s <.001) for each observer except G.J. and S.G. who showed very low and non-significant correlations. We performed a post-hoc analysis of tracking accuracy as a function of snapshot condition and whether or not the trial was reported as recognised (Figure 9). Observers Y.K and R.L. showed more accurate tracking for trials reported as recognised, as did observer M.L., although to a smaller and considerably overlapping degree. 

### Discussion

All observers demonstrated learning and showed full transfer of learning, except G.J., who showed only partial transfer. There was no significant difference between snapshot conditions for tracking accuracy. A group analysis employing signal detection theory seemed to indicate greater recognition of snapshots specified by observers compared to trials located near the end of a trajectory. However, individual analyses revealed this effect to be an artifact of poor recognition of portions of the end of a trajectory for observers G.J. and S.G. All other observers showed above chance recognition in each condition, with no difference between conditions. Thus, while there is again evidence that trajectory learning transfers between visual hemifields, there is no evidence that a strategy of identifying brief, more readily recognised portions of a trajectory aids in tracking.

## General Discussion

The aim of this study was to determine if hemifield independence of resources of attention is related to the relative dominance of attention versus memory processes, and if putative anatomical constraints on attention prevent between-hemifield transfer of improvements to tracking performance. This was evaluated by introducing an overt element of memory processing to an otherwise attention-driven task, by training observers to track targets with all objects displayed in one visual hemifield, and then testing if learning transferred from one hemifield to the other. If reduced hemifield independence is related to the dominance of memory processes over processes of attention, then hemifield independence was expected to be breached to some extent. The results of Experiment 1 suggested that this was the case; observers were able to learn to track trajectories displayed in one visual hemifield, and that learning transferred to the opposite hemifield, indicating an absence of a hemifield independence effect. Indeed for no observer was there a significant difference in performance in Experiment 1 for learned trajectories presented in the original hemifield as opposed to the opposite hemifield. Previous research has presented some evidence for hemifield independence of attention, although with particular restrictions; when tracking more than two targets in an MOT task [18], or when the task required detecting a change in location, but not colour [13]. This study extends the preceding research by demonstrating that hemifield independence does not apply to representations of learned trajectories.

It is possible that the task design of Experiment 1 might have allowed observers to rely on learning the end positions of objects rather than learning to track an entire trajectory. Experiment 2 investigated tracking under conditions that made the end positions of objects unreliable indicators of target locations. It was found that some inexperienced observers failed to show learning under these conditions, let alone transfer, indicating that some observers may indeed track objects by learning the trajectory end points. However, the results also showed that other observers could learn to track despite the trajectory end points being made unreliable. Experiment 3 aimed to evaluate a possible learning strategy, that of identifying and remembering brief snapshots, or moments of recognisable patterns of movement, and relying on recognition of those snapshots to aid tracking. The data did not reveal evidence of a reliance on snapshots, or of a reliance on recognition of objects near the end of a trajectory. This raises the question of what process does drive learning. Experiments 2 and 3 ruled out two possible strategies; memorising the final positions of objects, or memorising brief snapshots of movement. The current findings do not support the conclusion that learning is implicit [19,20]. Observers were able to recognise trained trajectory sets, and tracking accuracy was greater for trained than for new trajectory sets. These results demonstrate that observers were aware of what they learned, and that greater recognition was associated with greater tracking accuracy. We believe these findings warrant further investigation. In particular, it is unclear to what extent learning might be driven by both explicit and implicit elements. Further research might investigate this by directly comparing learning and recognition under structured training conditions such as those of this study, with performance under conditions of unstructured training of the kind employed in previous research [19]. It is worth noting that the design of the experiments may have allowed observers to become aware that learning was expected, as well as transfer of learning. In particular, one of the authors, M.L., was fully aware of the hypotheses of each experiment. However, M.L. showed partial transfer of learning in Experiments 1 and 2, and no advantage for snapshots in Experiment 3, while other observers showed full transfer. Therefore, any influence that awareness might have had did not enable M.L. to fully satisfy the hypotheses. Further, even if learning (transfer of learning) were dependent on an expectation of learning (or transfer), it is nonetheless evident that learning and transfer are possible.

The results of the current study also show that there are individual differences in the degree of learning in MOT, as could be expected given the individual differences in tracking ability [7]; however, the degree of learning an individual can achieve seems independent of the learning strategy they explicitly adopt. One observer showed relatively little learning despite moderately strong recognition, while two other observers showed a similar level of learning despite considerably different recognition accuracy, all while using the same strategy. Note that this may simply be due to implementing the strategy with varied effectiveness. The strategy of memorising the final positions of objects is clearly a poor strategy when the goal is to learn to track movement throughout a trial. Less obviously, the strategy of identifying short portions of a trial did not seem to influence learning, despite the strong intuition experienced by most observers that it was helpful. Regardless, there was strong evidence that trajectory learning can transfer from one visual hemifield to the other, lending support to a role for memory in overcoming putative anatomical constraints on spatial allocation of attention [12]. 

These results suggest that while resources of attention may be at least partially hemifield independent, memory resources are not [13]. However, it is unclear whether learning in MOT solely reflects improvement to processes of memory, or if attention is also improved. Previous research found that if distractor trajectories were repeated during training, and were then cued as targets during testing, tracking performance decreased [20]. This finding was interpreted as learned attentional suppression of distractors, and suggests one possible explanation for partial transfer of learning in some observers; if attentional resources are constrained to separate visual hemifields [12], or reflect greater interference between attentional foci within a visual quadrant compared with between quadrants [15], changes to attention in response to learning would not transfer between visual hemifields. On the other hand, memory resources are not constrained, and thus representations of trajectories would have been accessible regardless of the hemifield in which the stimuli appeared, thereby improving tracking despite the constraints on attention. Thus, difficulty in learning snapshots of a trajectory, where tracking performance is otherwise acceptable, is likely to reflect inadequate memory processing or resources rather than a failure of attention. Given the association between visual short-term memory and tracking performance [3,7], it is possible that individuals who show limited trajectory learning or transfer will also show limitations in visual short-term memory capacity or processing. Future research might also further help clarify the interaction between attention and memory by identifying the mechanisms involved in various aspects of learning in MOT. For example, if dynamic reallocation of attention during tracking [56] were found to improve in response to training, it would suggest that improving target-localisation is an important part of learning in MOT, and that the locus of such improvement is likely to be in processing of attention, not memory.

Research so far seems to agree that representations of MOT stimuli are comprised of spatiotemporal relationships between all objects in the scene, both targets and distractors [19,20,57]. However, these representations are not likely to be complete representations such as the frame-by-frame representation of a camera recording or digital animation. One alternative proposal is that the representations encode the motion paths of each object [19] although if this were the case, such encoding could not be averaged or summarised over time [57], implying a level of detail approaching that required for a frame-by-frame representation. This study investigated the possibility of a sparser version of a frame-by-frame representation, one that encodes brief portions of recognisable trajectories, rather than a complete encoding of an entire trial. The data do not provide evidence that recognition of portions of a trajectory contributes to tracking. 

Particular aspects of the results of the current study are consistent with statistical learning, while others are consistent with perceptual learning. It is worth noting that while the literature investigating perceptual learning and statistical learning has been relatively distinct, there are ongoing discussions regarding the relevant boundaries of both domains [36,58]. Regarding the current findings, encoding of spatial relationships between objects may be a form of statistical learning [20]. There is evidence that statistical learning occurs automatically and without awareness, although it does require attention [26,59]. In the current study recognition did appear to influence tracking, as discussed earlier. This suggests that learning in MOT is at least partially explicit under conditions similar to those of this study. However, it does not rule out the possibility that learning is also partially implicit, as previously demonstrated [19]. Indeed, the association between recognition and tracking accuracy shown here might be the result of increasing awareness of trajectory sets as they become easier to track. This would reflect an eventual awareness of what is being learned, despite the process of learning being implicit, and potentially continuing to proceed independent of awareness. It would be worthwhile investigating the rate at which recognition develops compared to accuracy when tracking repeated trajectory sets. If recognition develops later and more slowly than accuracy, that finding would lend support to the notion of learning in MOT being a form of statistical learning.

Statistical learning has been shown to transfer across space and time [60], a flexibility that has for a while been explicitly denied to perceptual learning [28]. However, there is now evidence that specificity of perceptual learning varies with task difficulty [61]. This influence of difficult on the specificity of perceptual learning may explain the partial transfer of learning displayed by some observers in the current study. In particular, those who showed partial transfer were also more likely to show less learning overall, consistent with the notion that greater task difficulty results in less transfer of learning. It should be noted that in the current study task difficulty was equated across observers, however this would not necessarily have controlled for differences in learning ability, nor does it provide any insight into which aspects of the task make learning more or less difficult. Further research might investigate the issues of task difficulty and difficulty of learning by, for example, varying task parameters such as the number of targets and distractors, the speed at which objects move, whether objects collide or pass over each other, and whether object motion is random or predictable. There is some evidence that the flexible allocation of cognitive resources can account for effects of set size, but not for effects of object speed and proximity [62]. Therefore, it is possible that variation in set size would influence learning in MOT to a greater extent than object speed or proximity.

The adaptive allocation of attention assumed to be occurring here is compatible with a flexible resource model of MOT. Iordanescu and colleagues [56] showed that observers are better able to precisely locate targets as distractors get closer to those targets, suggesting that more attention is being deployed to that location to enhance its spatial resolution. It seems likely that if attention can be reallocated on-demand, improvement of that process should be possible. Thus, a flexible resource model of MOT that incorporates on-demand reallocation of attention [45] would be likely to be suitable for modification to incorporate some aspects of learning in MOT. On-demand reallocation of attention is also compatible with models that propose increased uncertainty as a resource-dependent constraint on performance [62,63]. The models suggest that the resource in question might be either attention or memory. The current study suggests that both might have a role to play and provides further indication of how those roles might be better determined, by accounting for the effects of hemifield independence when tracking, and between-hemifield transfer of learning. One model, MOMIT [11], describes a role for VSTM and LTM that might also be extended to model effects of learning. However, as proposed the model does not account for the fact that some observers show learning and full transfer, while others show learning but only partial transfer. As discussed above, this is likely to involve changes to processes of attention as well as memory.

## Conclusion

In summary, this study investigated the interaction of attention and memory in MOT by introducing an overt element of memory processing to an otherwise attention-driven task. The aim was to determine if visual hemifield independence effects are related to the relative dominance of attention versus memory processes in a typical MOT task. We found that observers were able to learn to track unique MOT trajectories, and were able to transfer that learning between hemifields, without showing any hemifield independence effects. There was no evidence that learning was influenced by the recognition of brief portions of a trajectory. The findings lend support to a role for memory in overcoming putative anatomical constraints on the dynamic, distributed spatial allocation of attention involved in tracking multiple objects.
